# *In vitro* enrichment of trace elements promotes rapid germination of *Aspergillus* conidia: a clinical concern for immunosuppressed and hyperglycemic patients

**DOI:** 10.22034/cmm.2024.345251.1549

**Published:** 2024-11-22

**Authors:** Aishwarya Nikhil, Atul Kumar Tiwari, Ragini Tilak, Mohit Bhatia, Munesh Kumar Gupta

**Affiliations:** 1 Mycology Research Group, Department of Microbiology, Institute of Medical Science, Banaras Hindu University, Varanasi, India; 2 Department of Chemistry, Indian Institute of Technology (BHU), Varanasi, India; 3 Department of Tuberculosis and Chest, Sir Sunderlal Hospital (BHU), Varanasi, India

**Keywords:** Aspergillosis, Conidia germination, COVID-19, Diabetes, Immunocompromised patients, Trace elements

## Abstract

**Background and Purpose:**

This study aimed to examine the effects of essential trace elements, namely iron (Fe), manganese (Mn), zinc (Zn), and copper (Cu), combined with D-dextrose on conidial germination and growth of *Aspergillus fumigatus* and *Aspergillus flavus* ATCC strains. Trace elements are vital in metabolic processes, acting as cofactors for various enzymes; however, their precise role in fungal pathogenesis remains poorly understood.

**Materials and Methods:**

The research involved determining the minimum inhibitory concentrations (MIC) of Fe, Mn, Zn, and Cu for *Aspergillus* ATCC strains. Following MIC assessment, optimized concentrations of the trace elements (~140 and 550 pM) and various concentrations of D-dextrose (1-3% w/v) were introduced to assess their effects on fungal growth in RPMI 1640 broth. Growth was measured in terms of optical density, while conidial germination rates were also observed.

**Results:**

The MICs for Fe, Mn, and Zn were found to exceed 35 µM, while Cu exhibited lower MICs of 2 and 7.6 µM against *A. fumigatus* and *A. flavus*, respectively. At optimized concentrations, Fe, Mn, Zn, and Cu significantly enhanced fungal growth in both *Aspergillus* species. Additionally, growth rates increased proportionally with higher D-dextrose concentrations. Notably, the combination of enriched trace elements and D-dextrose resulted in up to 98% conidial germination.

**Conclusion:**

The findings demonstrate that optimized concentrations of essential trace elements and D-dextrose significantly promote conidial germination and growth of *Aspergillus* species *in vitro*. These results suggest that trace element supplementation might have important implications for immunocompromised and hyperglycemic patients. Further studies are warranted to explore the interactions between these micronutrients in fungal physiology and pathogenesis.

## Introduction

*Aspergillus* is a ubiquitous filamentous saprotroph that can cause life-threatening invasive fungal infections in immunosuppressed individuals [ 1 ]. *Aspergillus* conidia enter humans through inhalation and primarily affect the paranasal sinuses and lungs, where they manifest as severe asthma with fungal sensitization, allergic bronchopulmonary aspergillosis, sinus aspergillosis, chronic pulmonary aspergillosis, and invasive pulmonary aspergillosis [ 2 ]. Trace elements, such as iron, copper, zinc, and manganese, are essential micronutrients in humans, as iron is an integral component of hemoglobin [ 3 ]. Trace amounts of zinc are essential for immune response [ 4 ]. Moreover, copper (Cu) and manganese (Mn) are required for the function of nerves, connective tissues, and bone marrow [ 5 , 6 ]. Therefore, iron and zinc are frequently supplemented with occasional Mn [ 7 ]. In addition, glucose is essential for energy metabolism. Blood glucose levels are elevated in individuals with diabetes or those undergoing steroid therapy [ 8 ]. During the second wave of the severe acute respiratory syndrome coronavirus 2 (SARS-CoV-2) pandemic in India, a significant surge in respiratory aspergillosis cases was noted, manifesting as fever, cough with or without hemoptysis, breathing difficulty, and pleuritic chest pain [ 9 ].

This rapid increase in aspergillosis cases has been attributed to the use of iron and zinc supplementation, diabetes mellitus, or steroid-induced hyperglycemia [ 10 ]. Moreover, an association between COVID-19-associated mucormycosis and zinc supplementation has been reported in the literature [ 11 ]. Therefore, it is prudent to investigate the role of trace element supplementation in *Aspergillus* conidia germination. An *in vitro* study was performed to determine the enrichment effect of different trace elements, such as Fe, Mn, Cu, and Zn at variable concentrations, along with D-dextrose, on the germination behavior of *Aspergillus fumigatus* and *Aspergillus flavus* American Type Culture Collection (ATCC) strains.

## Materials and Methods

### Materials

Salt of trace elements (i.e., FeSO_4_, CuSO_4_, MnSO_4_, ZnSO_4_), and D-dextrose were procured from Sigma-Aldrich (USA). Fungal culture media potato dextrose agar (PDA), and RPMI 1640 (Roswell Park Memorial Institute, Buffalo, NY, USA) without Sodium bicarbonate with Morpholinepropane sulfonic acid (MOPS) buffer, and L-Glutamine were procured from Hi-Media Laboratories Limited (India). Other solvents and plastic wares were purchased from Merck and Tarson Product Private Limited (India). It should be mentioned that all reagents used were of analytical grade.

### Fungal strains, growth conditions, and harvesting of conidia

Lyophilized strains of *A. fumigatus* (ATCC 204305) and *A. flavus* (ATCC 204304) were acquired from ATCC (USA) and stored at -80 °C. The fungal strains were revived on PDA medium and incubated at 37 °C for 3-6 days. Freshly grown cultures were used for further experiments.

In this study, *A. fumigatus* and *A. flavus* were sub-cultured over a PDA slant and incubated at 37 °C for a week. Conidial harvesting was performed by the addition of 100 µL of Tween-20 (0.01% concentration), followed by the addition of 5 ml of normal saline with gentle shaking of the tubes. The resulting suspension was initially filtered using a vacuum membrane filter with a pore size of 0.45µm. The filtered suspension was pelleted at 4,000 rpm for 6 min and washed twice with PBS to remove media and surfactant traces. Subsequently, the conidial suspension was adjusted to an optical density (OD) of 0.08-0.1 at 565 nm to a solution containing 10^6^ conidia/ml.

### Antifungal susceptibility test of selected trace elements

Antifungal activities of the selected trace elements were evaluated by agar well diffusion against *A. fumigatus* and *A. flavus* strains, as described by Gizaw et al. [ 12 ]. Freshly harvested conidia of both strains were adjusted to 10^6^ conidia/mL. The prepared conidial suspension was swabbed over a freshly prepared Mueller-Hinton agar plate and left for 10 min to dry under a laminar hood. The wells were made in the swabbed Mueller Hinton agar with a sterile cork-borer and dispensed 50 µL of 100 nM voriconazole (positive control), 50 µL of distilled water (negative control), and 50 µL of 230 nM of each trace element salt (FeSO_4_, CuSO_4_, MnSO_4_, and ZnSO_4_) into each well. The plates were then incubated for 24 h at 37 °C, and the zone of inhibition was measured. The entire process was performed in triplicates.

### Minimal Inhibitory Concentration determinations of trace elements

Minimum inhibitory concentration (MIC) of the trace elements (Fe, Cu, Mn, and Zn) against *A. fumigatus* and *A. flavus* was determined by broth microdilution method using Clinical and Laboratory Standards Institute (CLSI) M38-A3 guidelines. Briefly, a 40 μM stock solution of each trace element salt was prepared and serially diluted by following a two-fold serial dilution method in a sterile 96-well flat-bottom microtiter plate containing 100 µL of RPMI-1640 along with MOPS buffer into each well, except the 1stand 11^th^ well. A 100 µl suspension of 103 conidia/ml in RPMI-1640 was poured into all wells except the 11^th^ well, followed by incubation at 37 °C for 48 h, and 100% growth inhibition was noted as the MIC value. The experiment was conducted in triplicate to ensure the reliability of the results. 

### Effect of trace elements and D-dextrose on Aspergillus growth by kinetic study

Effects of trace elements and D-dextrose (1-3% w/v) on the growth rate (depending on OD) of *A. fumigatus* and *A. flavus* were monitored. Initially, 230 µL of RPMI-1640 was dispensed into each well of a 96-well sterile flat-bottom microtiter plate, excluding the first column well. Subsequently, 10 µL of the trace element solution was added to each well, except for the first column well, which served as a control. Afterward, 250 µL of trace element solution was added to the control well, without the inoculum or RPMI-1640 broth. In addition, 10 µL of freshly harvested conidia of *Aspergillus* spp. was dispensed into each well, except the first column well. A well containing RPMI medium alone was used as the medium control. The same procedure was followed for D-dextrose with various concentrations (1-3% w/v). The OD was monitored for 8 h at 37 °C on a Multiskan Sky-High Plate Reader by recording the absorbance at 565 nm in triplicate. Subsequently, the mean OD value was calculated and plotted using Origin software (version 8.5).

### Effect of trace elements and D-dextrose on the germination rate of Aspergillus conidia

Effects of trace elements and D-dextrose on the conidial germination of *A. fumigatus* and *A. flavus* were evaluated. Briefly, 10^6^/ml conidia inoculums were prepared in RPMI-1640, as described above. This was followed by the addition of trace elements at concentrations of ~140 and 560 pM and D-dextrose (1-3% w/v) in a 15 ml sterile tube separately. Subsequently, the tubes were incubated at 150 RPM at 37 °C on an incubator shaker. Subsequently, at 0, 2, 4, 6, and 8 h of incubation, 50 µL of culture was withdrawn from each tube, and germination was observed under a bright-field compound light microscope (Olympus, Japan) at 400× magnification. The germination percentage of conidia was calculated using the following formula followed by statistical graph plotting:

Spores’ germination rate (%) = number of germinated conidia /total number of observed conidia

### Statistical analysis

*In vitro* experiments were used to investigate the effect of trace element supplementation on the growth rate of Aspergillus conidia. Non-parametric statistical tests were used to assess the data. The Spearman test was used to determine the correlation, and the Kruskal-Wallis and Friedman tests were used for column analysis. In addition, non-parametric two-way ANOVA was used for grouped analysis. Graphical representations of the kinetic study were created using GraphPad Prism software (version 8.4.2), and error bars indicate a 5% margin of error, when applicable. This approach enabled a scientifically rigorous assessment of the relationship between trace element supplementation and *Aspergillus* conidia growth rates, allowing for a better comprehension of the experimental results. *P* values of less than 0.05 were considered statistically significant in all tests.

## Results

### Antifungal activity of trace elements

**Figure 1:**
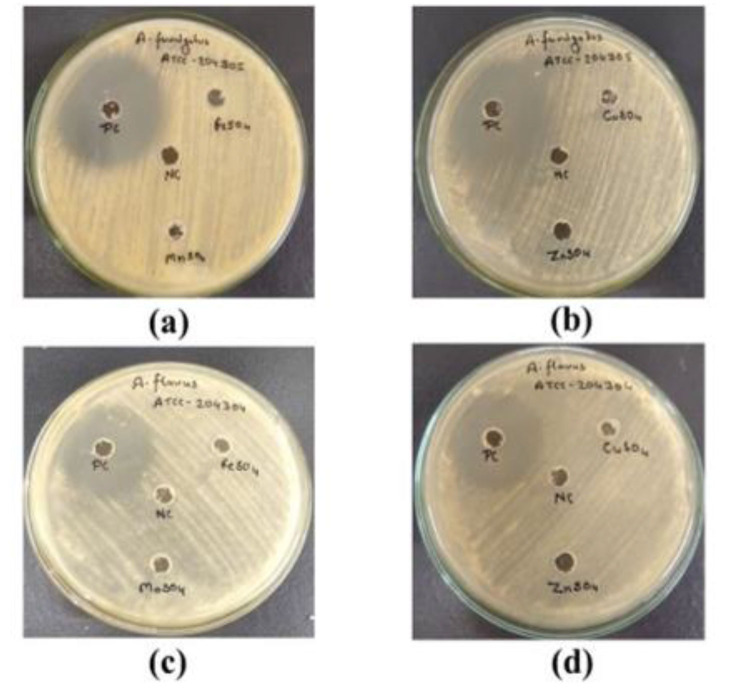
Antifungal evaluation of trace elements along with negative control (NC; Distilled Water) and positive control (PC; voriconazole) against *Aspergillus fumigatus* (ATCC 204305) and *Aspergillus flavus* (ATCC 204304). (a and b), FeSO_4_, MnSO_4_, CuSO_4_, and ZnSO_4_ against *A. fumigatus*; (c and d), FeSO_4_, MnSO_4_, CuSO_4_, and ZnSO_4_ against *A. flavus*.

This study evaluated the antifungal activity of the selected trace elements against *A. fumigatus* and *A. flavus*. The results demonstrated that all tested elements, namely Fe, Mn, Zn, and Cu, did not show any significant zone of inhibition whereas the positive control (voriconazole) showed zones of inhibition of 25 and 22 mm in *A. fumigatus* and *A. flavus*, respectively, as illustrated in Figure 1. Furthermore, the calculated MIC values of trace elements against *A. fumigatus* and *A. flavus* (as shown in Table 1) were more than 40 μM for FeSO_4_, ZnSO_4_, and MnSO_4_, while CuSO_4_ demonstrated an inhibitory effect at concentrations of 2 and 7.6 µM for *A. fumigatus* and *A. flavus*, respectively. 

**Table 1:** Minimum inhibitory concentration of trace elements against *Aspergillus fumigatus* and *Aspergillus flavus*

Trace elements	MIC against *A. fumigates* (ATCC 204305)	MIC against *A. flavus* (ATCC 204304)
FeSO_4_	>35Μm	>35μM
MnSO_4_	>35μM	>35μM
CuSO_4_	2μM	7.6μM
ZnSO_4_	>35 μM	>35 μM

ATCC: American Type Culture Collection

### Effect of trace elements over Aspergillus growth rate by kinetic study

**Figure 2:**
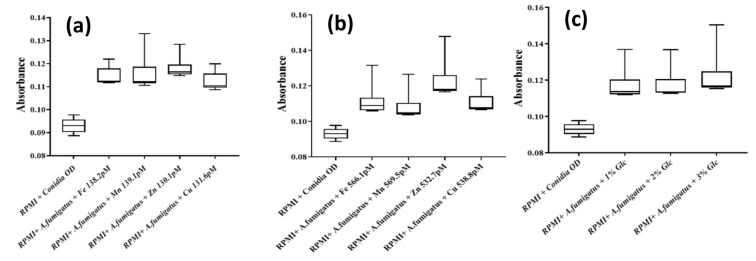
Whisker plot showing the effect of trace elements and D-dextrose supplementation for 8 h on the conidial overall growth (optical density) of *Aspergillus fumigatus* at high (Fe, 566; Mn, 569.5; Cu, 538.8 and Zn, 532.7 pM) and low concentrations (Kruskal-Wallis Statistics, Spearsman Correlation of X (time) value with each Y (trace element) value, Friedman test Statistics and two-way ANOVA; (*P*<0.0001).

**Figure 3:**
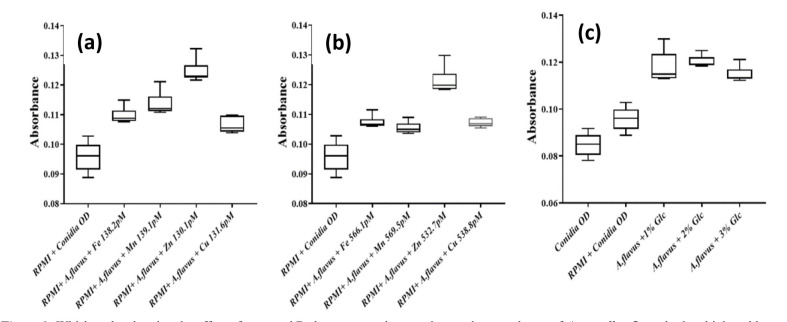
Whisker plot showing the effect of trace and D-dextrose supplementation on the growth rate of *Aspergillus flavus* both at high and low concentrations. (Kruskal-Wallis Statistics, Spearsman Correlation of X (time) value with each Y (trace element) value, Friedman test Statistics, and ordinary two-way analysis of variance (*P*<0.0001)

It was found that the addition of trace elements (FeSO_4_, MnSO_4_, CuSO_4_, and ZnSO_4_) significantly enhanced the growth of *A. fumigatus* and *A. flavus* after 8 h of incubation, compared to the control, as shown in figures 2 and 3. The increased growth was determined by comparison of the OD of RPMI alone with that of RPMI containing Aspergillus conidia with and without trace elements. Among these elements, Zn had a strong influence on the growth of *A. fumigatus* at a higher concentration of 532 pM, as shown in Figure 2b. However, at lower concentrations (≈140 pM), Fe and Mn had a stronger influence on growth than Zn and Cu, as shown in Figure 2. At lower concentrations (≈140 pM), the elements promoted conidial growth of *A. fumigatus* to Mn > Zn > Fe > Cu (Figure 2a); however, at higher concentrations (≈550 pM), elements influenced growth as Zn > Fe > Mn > Cu, as shown in Figure 2b. Moreover, the noted increase in growth rate was due to the enlarged size or germination of conidia. For this, a microscopic examination of the broth was performed, confirming Aspergillus conidia germination in the presence of different trace elements and D-dextrose. 

Furthermore, it has been noted that fungal conidia achieved the log phase shortly and were attributed to the rapid origin of germ tubes, compared to the control culture. Similarly, the conidial growth behavior of *A. flavus* was recorded after 8 h of incubation following enrichment with various concentrations (≈140 and 550 pM) of elements, as shown in Figure 3. Unlike *A. fumigatus*, *A. flavus* demonstrated significantly enhanced growth in the presence of Zn ions at both concentrations (130.1 and 532.7 pM). The remaining elements had the least effect on conidial growth, compared to the control conidial culture.

### Effect of D-dextrose over kinetic growth of Aspergillus species

Dextrose plays an indispensable role in the growth and pathogenesis of filamentous fungi, such as *Aspergillus* spp. Therefore, the effect of various concentrations of D-dextrose on *Aspergillus* conidial growth and germination was determined using kinetic analysis to understand the nutrient availability and growth behavior. Similar to the results obtained with trace element enrichment, the growth rate was enhanced after the addition of D-dextrose depending on the concentration. In *A. fumigatus*, 3% (w/v) D-dextrose enrichment demonstrated higher growth (25-30 %), compared to lower concentrations (1% and 2%), as shown in Figure 2c. In contrast, *A. flavus* showed enhanced growth (10-15%) at lower concentrations of 1% and 2% D dextrose, compared to 3% D-dextrose depicted in Figure 3c. 

### Evaluation of the influence of trace elements and D-dextrose on conidial germination rate

**Figure 4:**
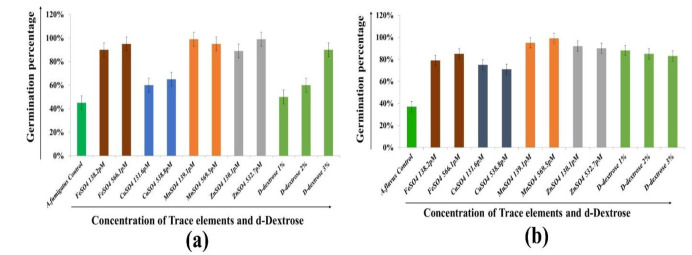
Graphical representation of conidial germination after the enrichment of variable concentration of trace elements and D-dextrose. (a) *Aspergillus fumigatus* and (b) *Aspergillus flavus*.

Trace elements (Fe, Mn, Cu, and Zn) are indispensable cofactors for housekeeping enzymes required for fungal growth and metabolism. Therefore, this study evaluated the influence of these elements on the conidial germination rate to correlate with the pathophysiology of Aspergillus conidia during infection in immunocompromised and hyperglycemic patients. The results demonstrated that germination in *A. fumigatus* conidia was variable in the presence of a predetermined concentration of trace elements and D-dextrose, as shown in Figure 4a.

However, *A. flavus* had a high germination rate in the presence of elements and D-dextrose, as shown in Figure 4b. Under *in vitro* conditions, conidial fungal germination did not follow a synchronous germination pattern. Therefore, it was also observed that only 40-50% of the conidia of *A. fumigatus* and *A. flavus* germinate after 8 h of incubation. Under *in vitro* conditions, fungal conidial germination did not follow a synchronous germination pattern. It was also observed that only 40-50% of the conidia of *A. fumigatus* and *A. flavus* germinated after 8 h of incubation. Under enrichment conditions, in this case of *A. fumigatus*, 98% Mn and Zn promoted conidial germination, followed by Fe (⁓94%) and Cu (50%), as shown in Figure 4a. After enrichment with 1%, 2%, or 3% D-dextrose, conidial germination increased by 10%, 20%, and 80%, respectively. Moreover, *A. flavus* conidia demonstrated maximum germination in the presence of Mn (98%) followed by Zn (90%) and Fe (88%). However, *A. flavus* had slow germination in the presence of Cu (78 % at a lower concentration), as depicted in Figure 4b. Overall, *in vitro* enrichment of selected trace elements and D-dextrose promoted the synchronous germination of *A. fumigatus* and *flavus* conidia within 8 h of incubation.

### Statistical Analysis

Data obtained from the *in vitro* supplementation of micronutrients from different growth rates of *Aspergillus* were analyzed using the SPSS software. Therefore, the correlation coefficient relationships between multiple independent variables were analyzed using Spearman’s correlation coefficient. Supplementation had a significant positive relationship with trace elements. This shows that trace element supplementation affects the germination of *Aspergillus* conidia. Statistical analysis was also performed using the Kruskal-Wallis test, Friedman test, and Two-way ANOVA of non-parametric tests using GraphPad Prism software (version 8.4). A non-parametric test was used since the data were distributed in numerical and categorical values, and it was more reliable with small sample sizes. The collected data were also less sensitive to outliers and extreme values with significance (*P*<0.05), indicating that micronutrient supplementation had a potential effect on the growth of *Aspergillus* spp. Statistical data are provided in the supplementary form.

## Discussion

Trace elements are indispensable nutrient factors required for the normal metabolism of fungal cells [ 13 ]. These elements are active centers of various enzymes and participate in the synthesis and metabolism of substances [ 14 ]. Moreover, elements have a significant influence on the stability of biological biomacromolecules and cell structures. They control the redox potential of cells and serve as an energy source for the proliferation of microorganisms [ 15 ]. Trace elements are also essential for the survival and germination of fungal conidia [ 16 ]. Furthermore, trace elements play a key role in host-fungal interactions [ 17 ]. 

The rationale behind the selection of trace elements was associated with observations during the second wave of SARS-CoV-2 infection in India (April 2021-July 2021) [ 18 ]. In the literature, supplementation of zinc and iron resulted in an increased incidence of mucormycosis and aspergillosis in hyperglycemic and immunosuppressed patients [ 19 ]. Therefore, it was prudent to investigate the relationship between the pathogenesis of *Aspergillus* and the supplementation of trace elements. Hence, the effects of four traces (viz. Fe, Cu, Mn, and Zn) on the conidial growth and germination of *A. fumigatus* and *A. flavus* were evaluated and a significant relationship was observed between trace element supplementation and germination of Aspergillus conidia. Increased conidial absorbance was observed in our kinetic study due to an increase in biomass, which revealed the active growth of conidia. The increased absorbance could be due to conidia metabolizing nutrients and undergoing cellular processes more actively after 8 h of incubation. 

Iron is a cofactor for catalase and peroxidase enzymes that neutralize cell-damaging reactive oxygen species (ROS) generated during fungal metabolism [ 20 ]. Moreover, fungal pathogens take up the supplemented iron through siderophores and utilize it in processes, such as DNA replication, chromatin remodeling, and mitochondrial respiration, thereby enhancing fungal growth [ 21 ]. Increased free iron has been reported as a predisposing risk factor for fungal infections. Therefore, to prevent fungal infections, the human host has a stringent regulatory mechanism that makes iron as inaccessible as possible to fungal conidia, especially during infection and accompanying inflammation, such as overproduction of hepcidin and the presence of natural iron chelators, both of which reduce free iron availability and fungal virulence and pathogenicity [ 20 ]. During SARS-CoV-2 infection, reduced hepcidin levels and hyperferritinemia were observed in patients with mucormycosis and aspergillosis [ 22 ]. Supplementation with iron played a significant role in fungal proliferation and growth, as demonstrated in the *in vitro* study depicted in Figure 4. Therefore, iron depletion or chelation therapy in SARC-CoV-2 patients might positively influence the control of these fungal infections [ 23 ]. Additionally, iron supplementation in the acute infective form of aspergillosis may be harmful. However, it should be noted that these results are based on an *in vitro* study. 

Moreover, Mn plays an indispensable role in the catalysis and functionality of various enzymes that regulate housekeeping biological processes, including oxidative phosphorylation, glycosylation, and signal transduction [ 23 ]. Specifically, Mn is required for fungal survival, growth, and virulence. In contrast, the human host utilizes nutritional immunity to sequester Mn to prevent the establishment of these fungal infections [ 23 ]. Naturally, neutrophils produce metal ion-binding calprotectin, which is secreted into the microenvironment to sequester essential metals from pathogens [ 24 ]. These strategic pathways significantly reduce the levels of Mn in the serum and tissues, protecting the host from invasion by fungal pathogens [ 25 ]. Moreover, Mn potentially influences the innate immune system of the human host by increasing myeloid cells and natural killer cell activity, thereby facilitating a strong defense against fungal infections [ 4 ]. However, under hyperglycemic and immunocompromised conditions, the sequestration of Mn from serum and extracellular fluids is impaired, resulting in easy access to Mn by the fungal pathogen Aspergillus. Additionally, external supplementation with Mn negatively affects the immune system. Compared to iron, Mn had a lower influence on the kinetic growth of *A. flavus*, while in *A. fumigatus*, a higher conidial germination rate (99%) was observed at lower concentrations, compared to the control (Figure 4). Overall, the presented data could be a decisive factor in the establishment of the relationship between Mn and fungal pathogenesis under immunocompromised conditions.

Similar to Fe and Mn, zinc is an important trace element, providing a favorable niche for fungal growth, as it regulates the DNA-binding proteins present in Class 3 zinc finger proteins (zinc cluster proteins). In addition, zinc also mediates fungal virulence by enhancing the production of gliotoxins in the phagocytic pathway, which hampers fungicidal activity [ 26 ]. Zinc-binding proteins, such as superoxide dismutase, generate ROS during host-pathogen interactions. Other zinc-binding proteins, such as aspartic proteases, serine proteases, and metalloproteases, cause tissue degradation, with subsequent fungal invasion and dissemination. In humans, zinc homeostasis is induced by the natural defense system, where specific membrane transporters or zinc-binding proteins regulate zinc uptake [ 27 ]. Zn intake was associated with fungal infections during the COVID-19 pandemic [ 28 ]. Chakravarty et al. reported Zn intake in 89.1% of patients with mucormycosis [ 21 ]. Moreover, reduced serum zinc levels have been reported to suppress fungal growth [ 29 ]. 

Findings of the present study support a previous report stating that zinc levels modulate the pathogenicity of *Aspergillus*. Therefore, the administration of trace amounts of zinc under *in vitro* conditions potently promoted the growth and conidial germination rate of both species of *Aspergillus*. Copper is a cofactor in the Cytochrome C oxidase enzyme, which is involved in respiratory chains, and plays a role in reductive iron assimilation, superoxide anion detoxifying superoxide dismutase, and melanin formation [ 30 ]. In the present study, the growth rate and germination underwent a significant enhancement with ~140 and 550 pM CuSO_4_. However, the MIC values of copper ions against *A. fumigatus* and *A. flavus* were 2 and 7.6 µM, respectively. This means that a lower concentration of Cu enhances fungal growth, whereas at higher concentrations, it reduces fungal growth.

Therefore, in this study, the effects of different concentrations of D-dextrose on the kinetic growth and conidial germination behaviors of *A. fumigatus* and *A. flavus* were also assessed. An enhanced growth rate was observed in proportion to sugar concentration, compared to 1% and 2% D-dextrose supplementation. A higher growth rate was observed with 3% D-dextrose. D-dextrose provides energy for conidial germination, as a combination of D-glucose and water has been reported to initiate conidial germination in *A. nidulans* (Osherov and May 2000) [ 31 ]. In addition, D-mannitol and D-trehalose are mobilized during the initial phases of germination, where D-trehalose metabolizes and produces d-glucose, which promotes germination [ 32 ]. Diabetic patients are 1.38-fold more prone to fungal infections since hyperglycemia induces fungal growth [ 33 ] and also raised sugar levels hamper the degranulation and neutrophil extracellular traps required for microbial killing [ 34 ]. In addition, higher sugar levels weaken natural barriers, thereby promoting fungal adhesion and invasion. It also results in increased ROS levels and pro-inflammatory cytokines, subsequently inhibiting phagocytic activity by affecting complement receptors [ 35 ]. 

All tested essential trace elements promoted *Aspergillus* conidia germination, even at low concentrations. Therefore, concentrated administration may promote the germination of *Aspergillus* conidia. This study also helped to determine the role of trace elements and D-dextrose in fungal pathogenesis, specifically in immunocompromised and hyperglycemic patients. However, the study was conducted *in vitro*; therefore, there is a need for *in vivo* correlation. Additionally, this study focused on the effect of trace elements on *Aspergillus* growth and germination, overlooking potential interactions with other factors in the complex host-fungal environment. There is also a need to study the interplay between trace elements and host immunity in fungal infections for future research.

## Conclusion

Based on the findings, *A. fumigatus* and *A. flavus* conidia achieve rapid log phase, grow faster, and enhance germination in the presence of a certain amount of trace elements (Fe, Zn, Cu, and Mn) under *in vitro* conditions. Similarly, D-dextrose pushes a proportionate kinetic growth and germination rate of *Aspergillus* spp. conidia in presence of 1-3% w/v.
